# A Calibration Protocol for Population-Specific Accelerometer Cut-Points in Children

**DOI:** 10.1371/journal.pone.0036919

**Published:** 2012-05-10

**Authors:** Kelly A. Mackintosh, Stuart J. Fairclough, Gareth Stratton, Nicola D. Ridgers

**Affiliations:** 1 Faculty of Education, Community and Leisure, Liverpool John Moores University, Liverpool, United Kingdom; 2 Research Institute for Sport and Exercise Sciences, Liverpool John Moores University, Liverpool, United Kingdom; 3 College of Human and Health Sciences, Swansea University, Swansea, Wales, United Kingdom; 4 School of Sports Science, Exercise and Health, The University of Western Australia, Perth, Australia; 5 Centre for Physical Activity and Nutrition Research, Deakin University, Victoria, Australia; University of Las Palmas de Gran Canaria, Spain

## Abstract

**Purpose:**

To test a field-based protocol using intermittent activities representative of children's physical activity behaviours, to generate behaviourally valid, population-specific accelerometer cut-points for sedentary behaviour, moderate, and vigorous physical activity.

**Methods:**

Twenty-eight children (46% boys) aged 10–11 years wore a hip-mounted uniaxial GT1M ActiGraph and engaged in 6 activities representative of children's play. A validated direct observation protocol was used as the criterion measure of physical activity. Receiver Operating Characteristics (ROC) curve analyses were conducted with four semi-structured activities to determine the accelerometer cut-points. To examine classification differences, cut-points were cross-validated with free-play and DVD viewing activities.

**Results:**

Cut-points of ≤372, >2160 and >4806 counts•min^−1^ representing sedentary, moderate and vigorous intensity thresholds, respectively, provided the optimal balance between the related needs for sensitivity (accurately detecting activity) and specificity (limiting misclassification of the activity). Cross-validation data demonstrated that these values yielded the best overall kappa scores (0.97; 0.71; 0.62), and a high classification agreement (98.6%; 89.0%; 87.2%), respectively. Specificity values of 96–97% showed that the developed cut-points accurately detected physical activity, and sensitivity values (89–99%) indicated that minutes of activity were seldom incorrectly classified as inactivity.

**Conclusion:**

The development of an inexpensive and replicable field-based protocol to generate behaviourally valid and population-specific accelerometer cut-points may improve the classification of physical activity levels in children, which could enhance subsequent intervention and observational studies.

## Introduction

There is need to establish children's physical activity levels for estimating prevalence, evaluating intervention effectiveness, and investigating relationships between physical activity and health [1]. However, physical activity in free-living situations is difficult to measure with precision as it encompasses a broad spectrum of behaviours and associated types of movement [2]. Accelerometry can enable the quantification of time spent at different activity intensities [3], [4] by applying pre-defined accelerometer count cut-points. There is though, large variation in the cut-points used to define children's moderate physical activity (MPA), vigorous physical activity (VPA) and sedentary time, which impacts on accurate estimation of physical activity levels [3]. To exemplify this, statistically significant differences in moderate-to-vigorous physical activity (MVPA) have been observed when MPA cut-points differ by as little as 90 counts•min^−1^ [5]. Thus, there is on-going debate concerning how to translate and interpret arbitrary accelerometer counts into more meaningful and interpretable units [6] that can be applied to specific study populations. Rather than researchers relying on empirically derived accelerometer cut-points that may not be appropriate to a given study sample, there is a need for behaviourally valid protocols that enable researchers to generate and apply cut-points that are relevant to specific research populations.

Though some field-based protocols have been used [7], [8], existing accelerometer cut-points have typically been generated using laboratory-based protocols [9], [10], allowing parallel measurement of energy expenditure (EE) by indirect calorimetry whilst controlling for physical activity intensity. Such methods however may hold limited ecological validity. Specifically, treadmill-based protocols have been used to obtain steady-state estimates of EE using a limited range of activities which do not capture intermittent lifestyle activities [11]. The result is that periods of intermittent physical activity may be erroneously coded as inactivity [12]. The unique nature of children's physical activity [13] warrants the development of behaviourally valid, population-specific accelerometer cut-points [4] which are cross-validated and evaluated using activities that are representative of children's free-living physical activity [4]. While researchers have identified the need for the development of straightforward, cost-effective calibration protocols [3], [4], the challenge remains to determine an appropriate sample of activities which represent the type and intensity of those performed by the target population [4]. Developing a field-based calibration technique that combines typical locomotor and free-play activities may replicate the diversity in children's natural physical activity participation [14] and help develop optimal population-specific physical activity thresholds [10].

The purpose of the present study was to develop and evaluate a field-based calibration protocol to create behaviourally valid and child population-specific accelerometer cut-point thresholds. Thus, a by-product of testing this protocol was new accelerometer cut-points which would be specific to the population under investigation, who were the focus of a subsequent school-based intervention [15]. With this in mind we emphasise that the aim was not to further saturate the research literature with more cut-points.

## Methods

### Ethics Statement

The study protocol was approved by Liverpool John Moores University Ethics Committee. Written assent from each subject and written informed signed consent from the primary caregiver were obtained. Participants were included in the study if they were without health problems which precluded their participation in usual daily physical activity.

### Participants

Twenty-eight children aged 10–11 years from one North-West England primary school participated in the study. Descriptive characteristics of the children are presented in Table 1.

**Table 1:** Participant Characteristics/

Characteristic	
Age (Years)	11.4±0.3
Height (m)	1.45±0.09
Body Mass (kg)	42.4±9.9
BMI	20.0±4.7
% Male	46.0%

### Protocol

Children completed 6 different activities to allow for both calibration and cross-validation (see Table 2 for a brief description). All activities were performed in a randomised order, and took place in the school playground or classroom as appropriate with 5 minutes seated rest between each activity. To capture both the sporadic nature of children's activity [13] and locomotor movements best suited to accelerometers [4], the activities incorporated both intermittent and continuous (i.e., walking and jogging) movements representative of culturally-relevant free-play situations. Sedentary activities were watching a DVD and drawing, which were consistent with those used previously [9].

**Table 2:** Descriptions of the activities performed by the children.

Activity	Location	Description
Drawing/Coloring*	Indoors	Child sat at a classroom table and was provided colored pencils, pencils, sharpener and paper and was asked to draw for 10 minutes in silence.
DVD Watching†	Indoors	Child sat at a classroom table and watched a DVD for 10 minutes in silence.
Self-paced Brisk Walking*	Outdoors	Child walked at their own pace around a circular track for 5 minutes but was asked to walk briskly at a pace that could be sustained for the whole 5 minutes.
Self-paced Jogging*	Outdoors	Child jogged at their own pace around a circular track for 5 minutes at a pace that could be sustained for the whole 5 minutes.
Playground Games*	Outdoors	For 10 minutes the child played 3 different playground games (see below) competitively with a member of the research team, with no breaks in-between each activity.
- Hopscotch		Child played hopscotch with a large dice on a playground drawn hopscotch for 3.3 minutes. Turns to hopscotch were alternate between participant and researcher.
- Frisbee		Child played Frisbee at their own pace across the playground for 3.3 minutes with the researcher.
- Reaction Ball		Child played reaction ball across the playground for 3.3 minutes with the researcher. The reaction ball is an oddly shaped ball that bounces in different directions when rolled. Therefore children had to react quickly to catch the ball.
Free-choice Games†	Outdoors	Child was provided with equipment; Frisbee, football, two tennis balls and rackets, two skipping ropes, two hula hoops, a reaction ball and a large dice, and were asked to play their choice of games, either on their own or with a member of the research team for 10 minutes. Participants were had to invite the researcher to play if they wanted and could freely change games throughout the 10 minutes.

* Calibration activity.

† Cross-validation activity.

### Instrumentation

#### Accelerometry

The ActiGraph GT1M (ActiGraph, LLC; Fort Walton Beach, FL) measures and records movement counts which reflect volume and intensity of physical activity. Prior to each testing session ActiGraphs were initialized (ActiLife 5.5.5; theActiGraph.com, Pensacola, FL) according to manufacturer specifications using 5-s epochs, to accurately capture the short duration, high frequency tempo of children's physical activity [16]. ActiGraphs were attached to an adjustable elastic belt that was fastened securely around the waist of the participant. The ActiGraph was positioned on the right mid-axilla line at the level of the iliac crest.

#### Direct Observation

Direct observation (DO) objectively captures the intermittent nature of children's physical activity [11] and has high internal validity [17]. The physical activity codes from the System for Observing Fitness Instruction Time (SOFIT) [18] were used to directly observe the children's physical activity behaviours during the activities. The physical activity coding element of SOFIT uses momentary time sampling to quantify health-related physical activity where codes 1 to 3 represented participants' body positions (lying down, sitting, standing), code 4 was walking, and code 5 (very active) was used for more intense activity than walking [18]. SOFIT was designed to assess physical activity during school physical education classes, but the same coding protocol has been used in other paediatric DO instruments to assess youth physical activity in settings such as the home (BEACHES; [19]), recreation centres, parks, and playgrounds (SOPLAY; [20]). These DO physical activity codes have been validated with heart rate monitoring [21], oxygen consumption [21], [22], and accelerometry [23], [24] with preschool to 12^th^ grade children, including those with developmental delays [17]. As a result, DO has been used as a criterion measure for validating other physical activity measures, and thus it is an appropriate method to underpin this calibration protocol which aims to objectively distinguish between different physical activity modes and intensities in children. Throughout the protocol each child's activity was coded every 10-s by a trained observer.

### Data Management

Prior to observation of each child, ActiGraphs and a digital watch were synchronized to allow data alignment. Following download of the data from the ActiGraph, ActiLife 5.5.5 (theActiGraph.com, Pensacola, FL) software was used to merge 5-s data to 10-s data in order to align mean activity counts with DO data. For each 10-s observation interval, DO codes were matched with the corresponding 10-s accelerometer counts. DO codes of 1 and 2 were categorized as sedentary time, code 3 as light intensity activity (LPA), 4 as MPA, and 5 as VPA.

### Statistical Analyses

Receiver operating characteristic (ROC) curve analyses were conducted using MedCalc 11.4.2.0 (MedCalc Software, Belgium) to determine cut-points for sedentary time, MPA, and VPA. ROC analysis determines the accuracy of a test or, in this case, a cut-point by examining the potential of the method to discriminate whether using the cut-point provides an accurate assessment of the activity intensity [25]. Essentially, the challenge is to determine a threshold that accurately captures “physical activity” (sensitivity) without capturing “inactivity” (specificity). The area under the ROC curve (AUC) is considered equivalent to the probability that a randomly drawn individual from the sample not meeting the criteria (e.g., MPA) has fewer accelerometer counts than those individuals who meet the cut-point criteria. Therefore the AUC is a measure of the accuracy of the cut-point. ROC AUC values of ≥0.90 are considered excellent, 0.80–0.89 good, 0.70–0.79 fair, and <0.70 poor [26].

ROC curve analyses were used with combined drawing (10 min duration), playground games (10 min duration), self-paced walking (5 min duration) and self-paced jogging activities (5 min duration). Mean accelerometer counts per 10-s of each calibration activity from all the participants were modelled as the independent variable. The dependent variable was calculated by creating a binary indicator variable based on DO, for the calibration activities. For MPA, DO codes of 1, 2, and 3 formed a binary code of 0, with codes 4 and 5 creating a binary code of 1. Similarly, for VPA DO codes of 1, 2, 3 and 4 formed a binary code of 0, with code 5 creating a binary code of 1. Finally, for sedentary behaviour DO codes of 1 and 2 created a binary code of 1, with DO codes 3, 4 and 5 being coded as 0. The sedentary and MPA cut-points provided the boundaries for the LPA classification. The ROC analyses identified the cut-points at which sensitivity and specificity were both maximized.

To examine classification differences and enable comparisons to previously published cut-points in this age-group, cut-points were cross-validated with the free-play and DVD viewing activities as recommended by Welk [4]. Two-by-two (2×2) contingency tables were used to check classification agreement. The observation and accelerometer data were first categorised into active and inactive binary codes. Computed sensitivity and specificity, Cohen's kappa coefficients [27], and percentage agreement between classifications were assessed. The determination of the optimal cut-point is a trade-off between sensitivity and specificity. It is not possible to speculate on the optimal balance between sensitivity and specificity, and so it is recommended that researchers consider the implications of their decisions regarding the selection of cut-points, by taking into account the impact on the outcome variable [11]. To highlight this contention, Guinhouya et al. [5] found statistically significant differences in the time spent in MVPA with ROC-derived cut-point differences of 90 counts•min^−1^, but suggested that a discrepancy of 200 counts•min^−1^ would be required for bio-behavioural relevance. Thus, we adjusted the calculated MPA threshold cut-point in our study by ±90, and ±200 counts•min^−1^ to evaluate the influence of such levels of variation on sensitivity, specificity, AUC, and cross-validation agreement.

## Results

**Figure 1:**
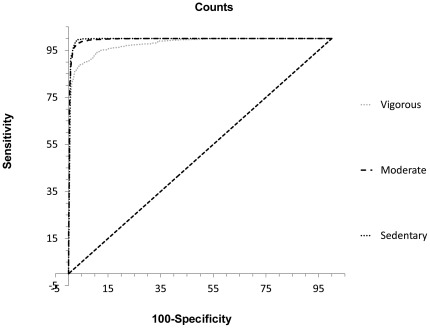
ROC curve for sedentary, moderate and vigorous.

The cut-points derived from the ROC analysis are shown in Table 3. Plots of the ROC curves are presented for sedentary, MPA and VPA (Figure 1). For all ROC analyses, the AUC was significantly better than chance with regards to global accuracy (*P*<.0001) and demonstrated excellent discriminatory power across activity intensities (.976–.995). The high specificity (95.8–97.4%) and sensitivity (88.7–99.2%) values indicate that the cut-points were unlikely to misclassify inactivity as activity, and that the cut-points were accurate in classifying periods of activity, respectively.

**Table 3:** ROC-derived cut-points for accelerometer counts per minute (CPM).

	CPM	Sensitivity	Specificity	AUC	CI
Sedentary	372	99.2%	96.9%	.995	.992–.996
Moderate	2160	96.9%	97.4%	.994	.992–.996
Vigorous	4806	88.7%	95.8%	.976	.972–.980

The classification agreement, sensitivity, specificity and kappa coefficient between calibration and cross-validation data for sedentary time, MPA, and VPA cut-points are shown in Table 4. The high percentage agreement (87.2–98.6%) and kappa scores (0.62–0.97) indicate that the cut-points were accurate in identifying periods of appropriate intensity. Also included for MPA were comparisons with the ROC-derived optimal cut-point ±90 and ±200 counts•min^−1^ [5] to reflect a compromise between sensitivity and specificity. Sensitivity and specificity values varied between 93–96% and 72–79% respectively, which illustrated the minimal trade-off resulting from manually challenging statistically optimal cut-points.

**Table 4:** Comparison of classification agreement, sensitivity, specificity, and kappa coefficients for different cut-points using cross-validation data (Free-play and DVD watching).

	CPM	Sensitivity	Specificity	Kappa	Agreement
*Sedentary*					
Optimized	372	98%	100%	.97	98.6%
*Moderate*					
Optimized	2160	94%	75%	.71	89.0%
Optimized +90	2250	95%	72%	.70	88.3%
Optimized −90	2070	94%	78%	.72	89.7%
Optimized +200	2360	96%	70%	.70	88.1%
Optimized −200	1960	93%	79%	.71	89.6%
*Vigorous*					
Optimized	4806	79%	89%	.62	87.2%

## Discussion

The primary aim of this study was to examine a straightforward field-based calibration protocol that could be used by researchers to define behaviourally valid, population-specific cut-points for sedentary time, MPA, and VPA. ROC curve analysis was used to identify criterion-referenced physical activity cut-points to apply to subsequent research which has sampled from the same population as this study. As advocated by Welk [4], the intention of this study was not to further saturate the literature with more cut-points, but to describe an objective, inexpensive, field-based protocol for population-specific calibration which could improve the precision of accelerometer thresholds in populations of interest.

Cut-points generated were ≤372, >2160 and >4806 counts•min^−1^, for sedentary time, MPA and VPA, respectively, which exhibited excellent classification accuracy [26]. In light of the growing body of evidence identifying sedentary time as an independent risk factor for a number of adverse health conditions [28], and risk markers such as insulin resistance [29], the generation of population-specific sedentary behaviour cut-points is essential. The present cut-point of ≤372 counts•min^−1^ for sedentary behaviour fell within the range of 100–799 counts•min^−1^ reported previously [7], [9], [29], [30]. Trost and colleagues [31] highlighted the tendency for waist-mounted accelerometers to misclassify static light-to-moderate intensity activities, (e.g., folding laundry and sweeping) as sedentary time, and this remains a legitimate concern. High sedentary cut-points may misclassify light intensity activity as sedentary and overestimate time spent in this behaviour (a false positive rate). Arguably 372 counts•min^−1^ is a relatively high sedentary cut-point and could therefore encompass LPA as well as sedentary time. Nonetheless, in accordance with a previous study [9], our protocol used drawing/colouring and DVD viewing as typical free-living sedentary activities, where children were seated. Despite Evenson and colleagues [9] reporting no significant differences in counts•min^−1^ between sitting still, watching a DVD and colouring, other studies [7], [30] found that incorporating activities whilst sitting was associated with markedly higher counts•min^−1^.

The current MPA cut-point of ≥2160 counts•min^−1^ was substantially lower than those reported by Treuth et al., [14] Mattocks et al., [32] Sirard et al. [7] and Puyau et al. [30] (range = 3000–3581 counts•min^−1^). According to Martinez-Gomez et al. [33] the use of different methodological protocols have consequently resulted in varying MVPA cut-points. A recent study by Trost et al. [31] recommended that researchers should use Evenson et al.'s [9] MVPA cut-point (≥2296 counts•min^−1^), which exhibited significantly better classification accuracy (sensitivity = 77%; specificity = 81%; AUC = 0.85) than other cut-points. Evenson et al. [9] were the only other authors to employ ROC analyses in a similar age group to that used in our study. Despite the similarity in derived MPA cut-points, Evenson and colleagues [9] incorporated three structured activities (stair climbing, brisk walking on a treadmill, dribbling a basketball). The use of semi-structured playground activities in the current study provided opportunities for children to be as active, or inactive, as they wanted, thus providing a range of counts. Furthermore, the protocol supported the intermittent nature of children's play incorporating a variety of activities. The present study demonstrated higher sensitivity (96.9%), specificity (97.4%) and AUC (0.99) for MPA values than Evenson et al. [9] suggesting that the protocol could efficiently generate population-specific cut-points in children. When applying Evenson et al.'s [9] MPA cut-points to our cross-validation data, there were little differences in the percentage agreement (88.2% vs. 89.0%, respectively) suggesting that both cut-points are robust.

The VPA cut-point of 4806 counts•min^−1^ was similar to those generated by Treuth et al. [14] and Sirard et al., [7] of 5200 and 5020 counts•min^−1^, respectively. Nonetheless, these cut-points are still higher than those reported in other work [6], [8], [9], [34], yet substantially lower than the values of 6130 and 8200 counts•min^−1^ developed by Mattocks et al. [32] and Puyau et al., [30] respectively. With the exception of the Sirard et al. [7] cut-points having a higher sensitivity value (95.8%), the present study was associated with higher sensitivity (88.7%), specificity (95.8%), and AUC (0.98), in comparison to values of 68.0–87.5%, 83.3–91.63%, and 0.83–0.97, respectively [7]–[9]. Specifically, when comparing the Evenson et al. [9] cut-points using our cross-validation data, the present study exhibited higher classification agreement (87.2% Vs. 83.1), demonstrating favourable sensitivity (79% Vs. 61%), suggesting that a higher VPA cut-point may be more appropriate in this specific population. Notably, the VPA cut-point exhibited lower classification accuracy than sedentary and MPA cut-points. As children's physical activity becomes more vigorous a larger associated range of movements (e.g., running, skipping, jumping, dodging, etc.) are performed and consequently there is more potential for variation within the counts. Additionally, the intermittent nature of children's physical activity in conjunction with the 10-s DO measurement period may lead to some movements being misclassified. Despite the potential error surrounding the VPA cut-point the classification accuracy was still excellent. Furthermore, excellent classification accuracy exhibited by all three cut-points suggests that the activities used in the protocol and the DO criterion measure were appropriate to effectively develop accurate population-specific cut-points for physical activity and sedentary behaviour.

The novel aspect of this study was the development of a pragmatic field-based protocol to develop population-specific cut-points, thus helping overcome issues surrounding cut-point selection. DO has frequently been cited as an appropriate criterion measure for evaluating children's physical activity [35]. The behaviour of the children was not controlled and incorporated free-choice activity. Therefore this field-based protocol holds strong ecological validity and may be more representative of children's physical activity behaviour than previous lab-based studies [9], [10]. The children performed a broad range of structured and unstructured activities and AUC (.976–.995) were considered to be accurate based on ROC criteria, indicating that the cut-points provided excellent discrimination across physical activity intensities [26]. This is an encouraging result considering the sporadic and intermittent nature of children's physical activity [13]. Moreover, the broad range of activities included in the protocol, combined with the excellent discrimination provides promise for minor modifications to the activities to accommodate cultural differences in children's physical activity modes.

The use of ROC analyses in the present study provided an objective balance between the needs for sensitivity and specificity, thus producing cut-points with maximal accuracy. Higher cut-point values tend to prioritise specificity over sensitivity, with the lower cut-points placing more emphasis on sensitivity. Without an empirical basis for the determination, it is difficult to select a trade-off between sensitivity and specificity. The volume of cut-points presented in the literature could have partly arisen as a result of the differential weight placed on sensitivity and specificity [11]. The ROC approach avoids this issue by placing equal importance on specificity and sensitivity in classification of activity by seeking to maximize the AUC.

This study had a number of strengths: (i) It used an ecologically sound, inexpensive field-based protocol to develop population-specific accelerometer cut-points representing sedentary behaviour, MPA, and VPA. Consistent with previous research [7] the activities included in the protocol resembled the usual free-living activities of children (i.e., watching a DVD, walking, and having free-choice of play); (ii) The use of ROC analysis facilitated comparisons of the relative sensitivity and specificity of the cut-points. We challenged the optimised sensitivity and specificity values for MPA by calculating respective values for the generated cut-point of 2160 counts•min^−1^ ±90 and ±200 counts•min^−1^ [5]. Results indicated that these adjustments had little effect on respective sensitivity and specificity values, suggesting that a degree of error exists around the cut-points. This may be due to each DO code being associated with a range of activity counts, which may explain some of the variation seen in the literature to date; (iii) Data were interrogated through cross-validation of the ROC-generated cut-points, which showed how gains in sensitivity are compensated by losses in specificity. The decision regarding what type of cut-point to use may depend on determining the most acceptable type of error for a particular research application. For example, intervention evaluations seeking to determine structured physical activity levels may need to emphasize specificity, thus indicating a reduced likelihood of classifying inactivity as activity (i.e., fewer false positives). Conversely, epidemiological studies on the health benefits of physical activity might be more effective with a cut-point that has higher sensitivity, preventing lower intensity activity from being missed (i.e., fewer false negatives).

Study limitations were: (i) The protocol included upper-body movements, which are not detected by hip mounted accelerometers [36]. However, as a relatively small proportion of movements are performed in this way compared to lower and whole body movements, the net effect is most likely small [37]; (ii) Anthropometrical and biomechanical factors such as stature, stride length, and body mass may have influenced accelerations detected by the accelerometer [7] during the protocol. Larger and more variable samples are needed to determine the effect of these factors on resultant cut-points; (iii) Though specific to this investigation the study sample size is small, however, the MPA and VPA intensity thresholds produced are similar to those detected through calibration research with larger samples [7], [9]; (iv) Even though AUC for the sedentary cut-point was high, the choice of sedentary activities may have incorporated some LPA, resulting in a relatively high cut-point. However, the protocol led to MPA and VPA cut-points in line with previous studies; (v) It is possible that gender differences in performance of some of the activities may have influenced accelerometer counts, though the sample size did not allow for gender-specific analyses. Preliminary inspection of the data however, indicated that gender differences were not evident, which concurs with previous research employing DO as a criterion measure of physical activity in similarly aged youth [11].

This novel study has demonstrated the potential utility of an ecologically sound, simple, inexpensive field-based protocol to derive optimal population-specific physical activity thresholds. In comparison to other studies adopting the ROC approach [7]–[10] the study demonstrated high sensitivity and specificity, and a high AUC for all three cut-points. The use of population-specific cut-points versus a single generic cut-point for children of varying age and demographics is a key methodological issue that has not been adequately addressed in the research literature. Collectively, our finding supports the application of a field-based calibration protocol to generate population-specific cut-points, though more work is required to generate a truly sedentary cut-point. This approach can be repeated in other populations to determine optimal physical activity thresholds for research, surveillance and programme evaluations. Without further research it is not possible to speculate on the optimal balance between specificity and sensitivity, so researchers should consider the implications of their decisions regarding the selection of cut-points. Our field-based protocol may help standardize accelerometry calibration approaches, reduce confusion generated through the plethora of reported cut-points and competing devices, and accommodate population-specific findings.

## References

[1] Sirard JR, Pate RR (2001). Physical activity assessment in children and adolescents.. Sports Med.

[2] Freedson PS, Miller K (2000). Objective monitoring of physical activity using motion sensors and heart rate.. Res Q Exer Sport.

[3] Jago R, Zakeri I, Baranowski T, Watson K (2007). Decision boundaries and receiver operating characteristic curves: new methods for determining accelerometer cutpoints.. J Sport Sci.

[4] Welk GJ (2005). Principles of design and analyses for the calibration of accelerometry-based activity monitors.. Med Sci Sport Exerc.

[5] Guinhouya CB, Lemdani M, Vilhelm C, Durocher A, Hubert H (2009). Actigraph-defined moderate-to-vigorous physical activity cut-off points among children: statistical and biobehavioural relevance.. Acta Paediatrica.

[6] Freedson PS, Pober D, Janz KF (2005). Calibration of accelerometer output for children.. Med Sci Sport Exerc.

[7] Sirard JR, Trost SG, Pfeiffer KA, Dowda M, Pate RR (2005). Calibration and evaluation of an objective measure of physical activity in preschool children.. J Phys Act Health.

[8] Van Cauwenberghe E, Labarque V, Trost SG, Bourdeaudhuji ID, Cardon G (2010). Calibration and comparison of accelerometer cut point in preschool children..

[9] Evenson KR, Catellier DJ, Gill K, Ondrak KS, McMurray RG (2008). Calibration of two objective measures of physical activity for children.. J Sport Sci.

[10] Alhassan S, Robinson TN (2010). Defining accelerometer thresholds for physical activity in girls using ROC analysis.. J Phys Act Health.

[11] Welk GJ, Eisenmann JC, Schaben J, Trost SG, Dale D (2007). Calibration of the Biotrainer Pro activity monitor in children.. Pediatr Ex Sci.

[12] Welk GJ, Corbin CB, Dale D (2000). Measurement issues for the assessment of physical activity in children.. Res Q Exerc Sport.

[13] Riddoch CJ, Mattocks C, Deere K, Saunders J, Kirkby J (2007). Objective measurement of levels and patterns of physical activity.. Arch Disease Chil.

[14] Treuth MS, Schmitz K, Catellier DJ, McMurray RG, Murray DM (2004). Defining accelerometer thresholds for activity intensities in adolescent girls.. Med Sci Sport Exerc.

[15] Mackintosh KA, Knowles ZR, Ridgers ND, Fairclough SJ (2011). Using formative research to develop CHANGE!: a curriculum-based physical activity promoting intervention.. BMC Public Health.

[16] Edwardson CL, Gorely T (2010). Epoch length and its effect on physical activity intensity.. Med Sci Sport Exerc.

[17] McKenzie TL (2010). 2009 C. H. McCloy Lecture. Seeing is believing: observing physical activity and its contexts.. Res Q Exerc Sport.

[18] McKenzie TL, Sallis JF, Nader PR (1991). SOFIT: System for observing fitness instruction time.. Journal of Teaching in Physical Education.

[19] Elder JP, Broyles SL, Mckenzie TL, Sallis JF, Berry CC (1998). Direct home observations of the prompting of physical activity in sedentary and active Mexican- and Anglo-American children.. J Dev Behav Pediatr.

[20] McKenzie TL, Marshall SJ, Sallis JF, Conway TL (2000). Leisure-time physical activity in school environments: an observational study using SOPLAY.. Prev Med.

[21] Rowe P, van der Mars H, Schuldheisz, J, Fox S (2004). Measuring students' physical activity levels: validating SOFIT for use with high-school students.. J Teach Phys Educ.

[22] Honas JJ, Washburn RA, Smith BK, Greene JL, Cook-Wiens G (2008). The System for Observing Fitness Instruction Time (SOFIT) as a measure of energy expenditure during classroom-based physical activity.. Pediatr Exerc Sci.

[23] Scruggs PW, Beveridge SK, Clocksin BD (2005). Tri-axial accelerometry and heart rate telemetry: relation and agreement with behavioral observation in elementary Physical Education.. Meas Phys Educ Exerc Sci.

[24] Sharma SV, Chuang R-J, Skala K (2011). Measuring physical activity in preschoolers: reliability and validity of the System for Observing Fitness Instruction Time for Preschoolers (SOFIT-P).. Meas Phys Educ Exerc Sci.

[25] Zweig MH, Campbell G (1993). Receiver-operating characteristic (ROC) plots: a funamental evaluation tool in clinical medicine.. Clin Chem.

[26] Metz CE (1978). Basic principles of ROC analysis.. Semin Nucl Med.

[27] Cohen J (1960). A coefficient of agreement for nominal scales.. Educ Psych Meas.

[28] Owen N, Healy GN, Matthews CE, Dunstan DW (2010). Too much sitting: the population health science of sedentary behavior.. Exerc Sport Sci Rev.

[29] Ekelund U, Anderssen SA, Froberg K, Sardinha LB, Andersen LB (2007). Independent associations of physical activity and cardiorespiratory fitness with metabolic risk factors in children: The European Youth Heart Study.. Diabetologia.

[30] Puyau MR, Adolph AL, Vohra FA, Butte NF (2002). Validation and calibration of physcial activity monitors in children.. Obes Res.

[31] Trost SG, Loprinzi PD, Moore R, Pfeiffer KA (2011). Comparison of accelerometer cut-points for predicting activity intensity in youth.. Med Sci Sport Exerc.

[32] Mattocks C, Leary S, Ness AR, Deere K, Saunders J (2007). Calibration of an acclerometer during free-living activities in children.. Int J Pediatr Obes.

[33] Martinez-Gomez D, Ruiz JR, Francisco BO, Sjöström M (2011). Author Response.. Am J Prev Med.

[34] Pate RR, Stevens J, Pratt C, Sallis JF, Schmitz KH (2006). Objectively measured physical activity in sixth-grade girls.. Arch Pediatr Adolesc Med.

[35] McKenzie TL, Welk GJ (2002). Use of direct observation to assess physcial activity.. Physical Activity Assessments for Health-Related Research.

[36] Rowlands AV, Thomas PW, Eston RG, Topping R (2004). Validation of the RT3 triaxial accelerometer for the assessment of physical activity.. Med Sci Sport Exerc.

[37] Welk G (2002). Physical Activity Assessments for Health-Related Research..

